# Refining microbial community metabolic models derived from metagenomics using reference-based taxonomic profiling

**DOI:** 10.1128/msystems.00746-24

**Published:** 2024-08-13

**Authors:** Marwan E. Majzoub, Laurence D. W. Luu, Craig Haifer, Sudarshan Paramsothy, Thomas J. Borody, Rupert W. Leong, Torsten Thomas, Nadeem O. Kaakoush

**Affiliations:** 1 School of Biomedical Sciences, Faculty of Medicine and Health, UNSW Sydney, Sydney, New South Wales, Australia; 2 School of Clinical Medicine, Faculty of Medicine and Health, UNSW Sydney, Sydney, New South Wales, Australia; 3 Department of Gastroenterology, St. Vincent’s Hospital, Sydney, New South Wales, Australia; 4 Concord Clinical School, University of Sydney, Sydney, New South Wales, Australia; 5 Department of Gastroenterology, Concord Repatriation General Hospital, Sydney, New South Wales, Australia; 6 Centre for Digestive Diseases, Sydney, New South Wales, Australia; 7 Centre for Marine Science and Innovation, School of Biological, Earth and Environmental Sciences, Faculty of Science, UNSW Sydney, Sydney, New South Wales, Australia; CNRS Delegation Bretagne et Pays de Loire, Nantes, France

**Keywords:** metabolic modelling, microbiome, human, environmental, metagenomics

## Abstract

Characterization of microbial community metabolic output is crucial to understanding their functions. Construction of genome-scale metabolic models from metagenome-assembled genomes (MAG) has enabled prediction of metabolite production by microbial communities, yet little is known about their accuracy. Here, we examined the performance of two approaches for metabolite prediction from metagenomes, one that is MAG-guided and another that is taxonomic reference-guided. We applied both on shotgun metagenomics data from human and environmental samples, and validated findings in the human samples using untargeted metabolomics. We found that in human samples, where taxonomic profiling is optimized and reference genomes are readily available, when number of input taxa was normalized, the reference-guided approach predicted more metabolites than the MAG-guided approach. The two approaches showed significant overlap but each identified metabolites not predicted in the other. Pathway enrichment analyses identified significant differences in inferences derived from data based on the approach, highlighting the need for caution in interpretation. In environmental samples, when the number of input taxa was normalized, the reference-guided approach predicted more metabolites than the MAG-guided approach for total metabolites in both sample types and non-redundant metabolites in seawater samples. Nonetheless, as was observed for the human samples, the approaches overlapped substantially but also predicted metabolites not observed in the other. Our findings report on utility of a complementary input to genome-scale metabolic model construction that is less computationally intensive forgoing MAG assembly and refinement, and that can be applied on shallow shotgun sequencing where MAGs cannot be generated.

**IMPORTANCE:**

Little is known about the accuracy of genome-scale metabolic models (GEMs) of microbial communities despite their influence on inferring community metabolic outputs and culture conditions. The performance of GEMs for metabolite prediction from metagenomes was assessed by applying two approaches on shotgun metagenomics data from human and environmental samples, and validating findings in the human samples using untargeted metabolomics. The performance of the approach was found to be dependent on sample type, but collectively, the reference-guided approach predicted more metabolites than the MAG-guided approach. Despite the differences, the predictions from the approaches overlapped substantially but each identified metabolites not predicted in the other. We found significant differences in biological inferences based on the approach, with some examples of uniquely enriched pathways in one group being invalidated when using the alternative approach, highlighting the need for caution in interpretation of GEMs.

## INTRODUCTION

Microbial communities perform essential functions within their host, such as stimulating and developing the host immune system, providing protection against pathogens, maintaining metabolic homeostasis, and generating nutrients through pathways that do not exist in the host (1–3). The host-associated microbiome can perform these functions through the production of a range of metabolic products including short-chain fatty acids, secondary and tertiary bile acids, and by-products of tryptophan metabolism among many others (4–8). Moreover, microbial communities perform essential ecological functions within their environmental niches such as biogeochemical cycling (9, 10). Thus, there is a growing interest in deciphering the metabolic output of microbial communities.

Metabolic network modeling is a system biology approach that enables the inference of metabolic outputs from genomic information (11). The construction of genome-scale metabolic models (GEMs) has provided researchers with the ability to study strain- and environment-specific metabolism (12–14). Modeling can also be expanded from individual strains to a community level through several ways that are still subject to refinement (15). These *in silico* models provide a platform for researchers to predict metabolic interactions that occur within a microbial community, as well as metabolic activities under specific environmental conditions (16, 17). Thus, a GEM can allow for the prediction of pathways that may be important to the host or environment and optimal culture conditions for lesser known microorganisms (15).

When constructing a community-level GEM from metagenomes, one approach is to input metagenome-assembled genomes (MAGs); however, input of reference genomes based on taxonomic profiling has also been proposed (18). Advances in the sequencing technology has enabled deeper profiling of microbial communities through shotgun metagenomics and the capacity for production of more complete MAGs that subsequently improve the quality of GEMs. These technological advances have also led to a substantial increase in coverage of reference pangenomes for bacterial species, providing an opportunity to utilize these pangenomes in GEM construction. To date, little is known about the influence of the genomic input strategy on community-level metabolic models based on metagenomes.

Here, we constructed GEMs using shotgun metagenomic data from human stool samples as well as environmental samples (seawater and sediment) by using high- and medium-quality MAGs as input (MAG-guided approach) and, in parallel, constructed GEMs using an approach guided by species relative abundances calculated from taxonomic profiling (reference-guided approach). We validated inferred outputs from the human samples using longitudinal untargeted metabolomics data. We then compared the efficiency and differences in biological inferences of the two approaches when applied to microbiotas with different efficacies when used to treat patients with ulcerative colitis.

## RESULTS

### Selection of features from human metagenomic samples for modeling

**Fig 1:**
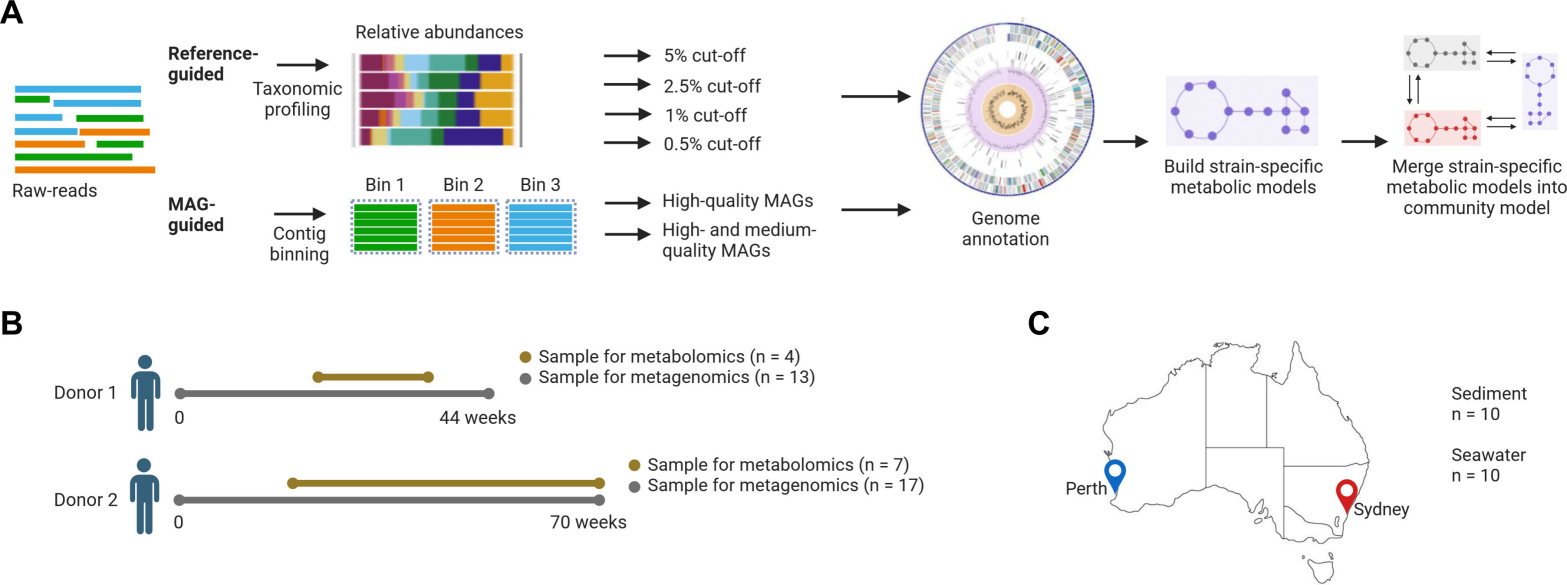
Metabolic modeling pipeline and study samples. (**A**) Framework for the community metabolic modeling using the reference-guided and MAG-guided approaches. (**B**) Sampling from the two healthy individuals for the metagenomics and metabolomics. (**C**) Sampling sites for the environmental metagenomic samples.

The taxonomic composition within each human stool sample was profiled using MetaPhlAn4, and relative abundances of microbial species were determined (Fig. 1A and B). Mean relative abundances of species per individual (i.e., donor 1 or 2) were calculated based on all available samples from that donor. Species with mean relative abundances of greater than or equal to 5%, 2.5%, 1%, or 0.5% were selected for the reference-guided approach as four independent inputs per donor (Fig. 1A; Table 1). In addition, MAGs assembled from the same samples were classified as either high quality or medium quality and selected as two independent inputs per donor for the MAG-guided approach (Fig. 1A; Table 1). Thus, six predictions identifying the net metabolic output of the community were made for each donor microbiota using these reference-guided and MAG-guided approaches (Fig. 1A).

**TABLE 1:** Microbial community metabolic modeling in human fecal samples for the reference-guided and MAG-guided approaches^*a*^

Donor	Parameter	Reference-guided				MAG-guided		
		5%	2.5%	1%	0.5%	High	High and medium	Normalized
Donor 1	Taxa	6	10	20	34	56	86	34
	Total predicted metabolites	5,568	9,149	17,812	28,583	44,928	67,924	28,172
	Total extracellular metabolites	131	145	184	200	191	196	185
	Unique metabolites	1,248	1,290	1,402	1,500	1,519	1,543	1,493
	Confirmed unique metabolites (total)	167	170	180	183	184	185	183
	Data loss (%)	31.41	30.62	30.03	30.13	30.22	30.33	30.21
	Confirmed unique metabolites (%)	19.51	18.99	18.35	17.46	17.36	17.21	17.56
Donor 2	Taxa	2	6	24	37	67	84	37
	Total predicted metabolites	1,910	5,570	21,318	33,661	55,818	69,840	31,902
	Total extracellular metabolites	102	148	176	219	223	223	221
	Unique metabolites	1,062	1,251	1,404	1,562	1,576	1,579	1,545
	Confirmed unique metabolites (total)	145	154	171	180	180	180	180
	Data loss (%)	27.50	31.73	30.77	30.15	30.71	30.72	30.36
	Confirmed unique metabolites (%)	18.83	18.03	17.59	16.50	16.48	16.45	16.73

^*a*^ Summary includes the number of input bacterial species used for the metabolic predictions for each donor (taxa), the total number of predicted metabolites (duplicates not removed), total number of metabolites classified as extracellular (duplicates not removed), the number of non-duplicated total predicted metabolic products (unique metabolites), and the number of metabolites validated using untargeted metabolomics (confirmed unique metabolites). Data loss resulted from the predicted metabolic product not having an assigned Kyoto Encyclopedia of Genes and Genomes ID that can be matched to the untargeted metabolomics output and no standardized naming convention.

The species selected at different relative abundance thresholds for input (*n* = 6, 10, 20, and 34) into the reference-guided approach for donor 1 (Table 1; Table S1) were classified to a total of 23 genera that included several unnamed genus-level genomic bins that can be obtained from Pasolli et al. (19). Selected species for donor 2 (*n* = 2, 6, 24, and 37) included bacteria belonging to 24 genera. For the MAG-guided approach, a total of 56 high-quality MAGs and 86 medium- or high-quality MAGs as well as 67 high-quality MAGs and 84 medium- or high-quality MAGs were assembled from donor 1 and donor 2 samples, respectively (Tables S2 and S3). The analysis included MAGs from 70 and 65 genera for donors 1 and 2, respectively. The total number of uniquely classified taxa that were inputted into the GEMs was higher (>2-fold) in the MAG-guided approach than the reference-guided approach (i.e., high + medium vs 0.5% cutoff) (Table 1). Metabolite predictions from individual taxa inputted into the models, including the percentage of blocked reactions, are provided in Table S4.

### Outcomes of metabolic predictions in human metagenomic samples

A total of 5,568, 9,149, 17,812, and 28,583 metabolic compounds were predicted for donor 1, while a total of 1,910, 5,570, 21,318, and 33,661 were predicted for donor 2 using the 5%, 2.5%, 1%, and 0.5% relative abundance cutoffs, respectively (Table 1). These included a total of 1,248 and 1,062 unique metabolic compounds predicted for donor 1 and donor 2 samples, respectively, for the reference-guided approach when a relative abundance cutoff of 5% was implemented (Table 1). The total number of unique metabolic compounds further increased to 1,290, 1,402, and 1,500 for donor 1 and 1,251, 1,404, and 1,562 for donor 2 when the relative abundance cutoffs of 2.5%, 1%, and 0.5% were employed (Table 1). The number of novel predicted metabolites appeared to saturate with increasing number of input taxa (Fig. S1A), which is likely due to metabolic redundancy. In comparison, despite a higher number of input taxa and a higher number of total predicted metabolites (Table 1), 1,519 and 1,576 unique metabolic compounds were predicted in donor 1 and donor 2 samples, respectively, using the high-quality MAGs alone, while a total of 1,543 and 1,579 unique metabolic compounds were predicted in donor 1 and donor 2 samples, respectively, using both high- and medium-quality MAGs (Table 1). Thus, while the reference-guided approach predicted substantially lower total numbers of metabolites, it predicted a similar number of unique metabolites in both donors with less input taxa (i.e., 1,500 from 34 for donor 1 and 1,562 from 37 for donor 2 vs 1,543 from 86 for donor 1 and 1,579 from 84 for donor 2), possibly due to MAGs being incomplete. We tested this by normalizing the number of input taxa in the MAG-guided approach to the reference-guided approach (34 and 37 most complete high-quality MAGs from donor 1 and donor 2, respectively), showing a lower number of total and unique predicted metabolites (Table 1).

**Fig 2:**
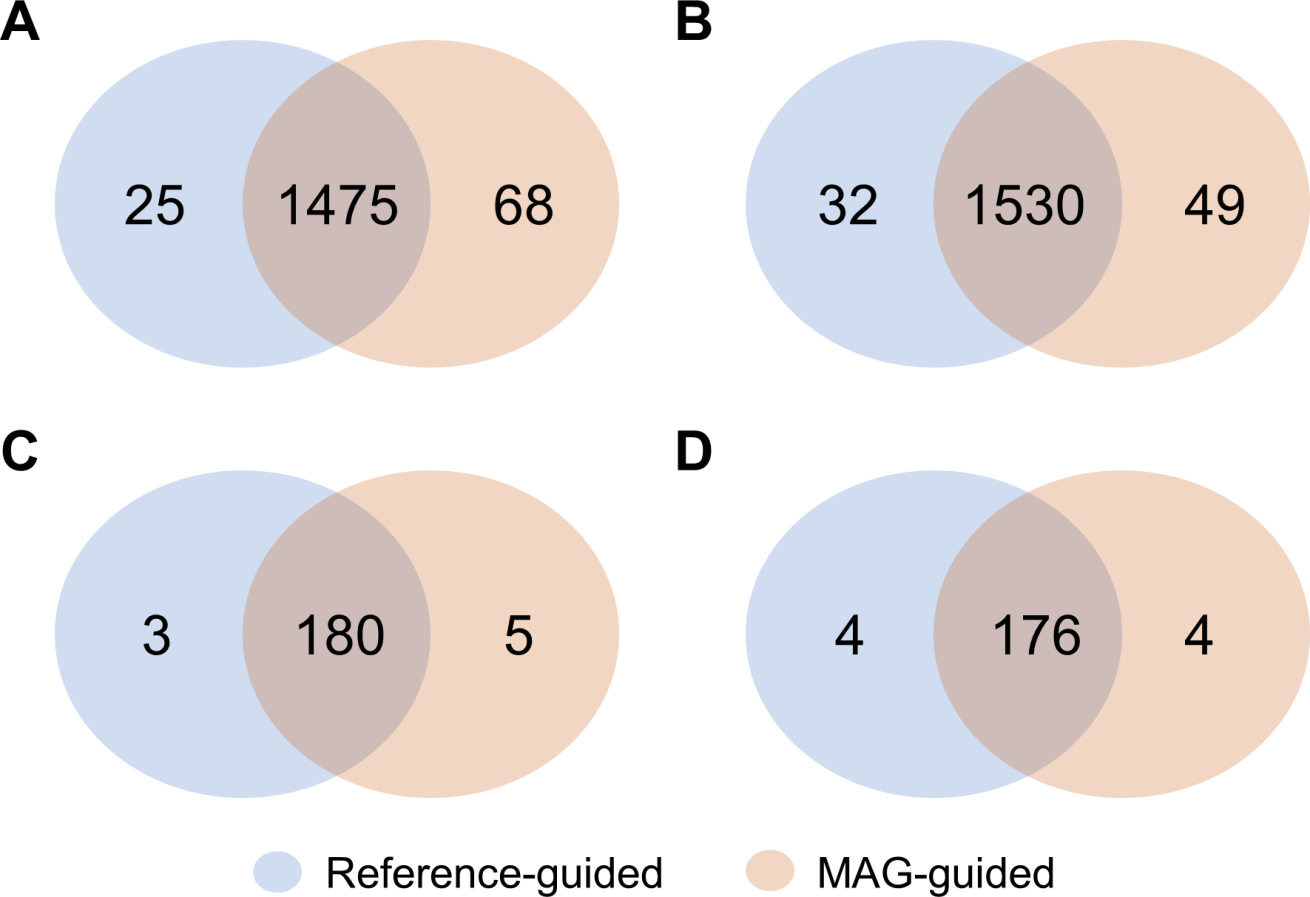
Number of unique metabolites across the reference-guided (0.5% cutoff) and MAG-guided (high- and medium-quality) inputs for human fecal samples. (**A**) Predicted metabolites for donor 1. (**B**) Predicted metabolites for donor 2. (**C**) Confirmed metabolites for donor 1. (**D**) Confirmed metabolites for donor 2.

Next, we assessed if the two approaches were additive or complementary (Fig. 2A and B; Fig. S2). A substantial amount of overlap was observed across the two approaches (*n* = 1,475 of 1,543 and 1,530 of 1,579 for donor 1 and donor 2, respectively), with reference-guided approach predicting 25 and 32 unique metabolites for donor 1 and donor 2, respectively (Fig. 2A and B; Fig. S2). The MAG-guided approach also predicted unique metabolites not predicted by the reference-guided approach (68 and 49 for donor 1 and donor 2, respectively) (Fig. 2A and B; Fig. S2).

### Validation of metabolic predictions in human metagenomic samples with untargeted metabolomics

To validate metabolite predictions from the community models from the reference-guided and MAG-guided approaches, they were compared against untargeted metabolomics data generated longitudinally in each donor. A total of 167 and 145 metabolic products were validated in donor 1 and donor 2 samples, respectively, for the reference-guided approach when a relative abundance cutoff of 5% was implemented (Table 1). The total number of validated non-redundant metabolic products further increased to 170, 180, and 183 for donor 1 and 154, 171, and 180 for donor 2 when the relative abundance cutoffs of 2.5%, 1%, and 0.5% were employed (Table 1). A total of 184 and 180 metabolic products were validated in donor 1 and donor 2 samples, respectively, using the high-quality MAGs alone, while a total of 185 and 180 metabolic products were identified in donor 1 and donor 2 samples, respectively, using both high- and medium-quality MAGs (Table 1). Overall, a similar number of non-redundant metabolites were validated using the reference-guided approach and the MAG-guided approach (Table 1). Data loss, resulting from a lack of Kyoto Encyclopedia of Genes and Genomes (KEGG) IDs for a portion of predicted metabolites and non-standardized naming conventions, was similar in both the reference-guided data and MAG-guided data (Table 1). Both approaches had a similar susceptibility to error given that similar percentages of total unique metabolites were validated across both donors (Table 1). A further important point is that we did not calculate accuracy, specificity, and sensitivity due to our ability to only validate non-redundant metabolites rather than the total predicted metabolic output of the community, which we believe confounds the calculation of these parameters.

We then assessed if the approaches were complementary or additive for the confirmed metabolites (Fig. 2C and D). For donor 1, five unique metabolites in the MAG-guided approach were not predicted by the reference-guided approach. In contrast, three validated metabolites were predicted by the reference-guided approach but not the MAG-guided approach. Similarly, in donor 2, the MAG-guided approach predicted four metabolites unique to it while the reference-guided approach also predicted four metabolites unique to it, highlighting that there is substantial overlap across the approaches.

### Changes to biological inferences based on the selected approach

To establish differences in biological inference gained by the selected approach, we performed pathway enrichment analysis across the different predicted unique metabolites lists. We first assessed within-donor variation by determining the significantly enriched pathways (*q* < 0.05) for metabolite outputs from each approach (Table S5). We identified 141 and 147 pathways to be common across the approaches for donor 1 and donor 2, respectively, whereas a total of 10 and 4 were unique to one approach (Table S5). We then compared the significantly enriched pathway outputs from each of the donor 1 and donor 2 microbiotas (Table S6). The microbiota from these donors have been shown to have variable efficacies (100% vs 36.4% for *n* = 15 patients, Fisher’s *P* = 0.026) in the context of treating patients with ulcerative colitis (20), making the identification of true unique metabolic pathways of therapeutic relevance. When using the reference-guided approach, five and seven pathways were uniquely enriched in donor 1 and donor 2, respectively. In contrast, only one and five pathways were uniquely enriched in donor 1 and donor 2 when using the MAG-guided approach. Notably, lysine degradation (SMP00037) was identified to be uniquely enriched in donor 1 in the reference-guided approach, whereas the KEGG version of lysine degradation (map00310) was uniquely enriched in donor 2 using the MAG-guided approach. This highlighted the variation in biological inferences depending on the approach employed.

To determine if biological inferences change further when combining outputs from the two approaches, we merged the predicted metabolite lists from the reference- and MAG-guided approaches, removed duplicate metabolites, and then compared pathway enrichment within and between the two donors. This resulted in 148 and 149 pathways to be significantly (*q* < 0.05) enriched in donor 1 and donor 2, respectively (Table S7). For donor 1, the 148 pathways corresponded to 131 common across approaches, 3 specific to the reference-guided approach, 14 specific to the MAG-guided approach. For donor 2, the 149 pathways corresponded to 144 common across approaches, two specific to the reference-guided approach, two specific to the MAG-guided approach, and one novel pathway, highlighting that within-donor inferences can change. For the comparison between donors using the combined data, two and three enriched pathways were identified to be unique to each donor at *q* < 0.05, with the only result found to be consistent across the reference-only, MAG-only, and combined approaches being enrichment of propanoate metabolism in donor 2 (Table 2; Table S7). Furthermore, biosynthesis of terpenoids and steroids (map01062), initially shown to be uniquely enriched in donor 1 using both approaches, was identified in donor 2 when using the combined approach (Tables S6 and S7).

**TABLE 2:** Metabolic pathways significantly enriched only in donor 1 or donor 2 using the combined non-redundant metabolic prediction outputs from both approaches

Donor	Pathway ID	Pathway name	Source	*q*-value
Donor 1	SMP00037	Lysine degradation	HMDB	0.031
	map00562	Inositol phosphate metabolism	KEGG	0.046
Donor 2	SMP00016	Propanoate metabolism	HMDB	0.0071
	SMP00020	Arginine and proline metabolism	HMDB	0.021
	SMP00450	Phytanic acid peroxisomal oxidation	HMDB	0.034

### Outcomes of metabolic predictions in environmental samples

To determine if the differences in metabolite predictions between the two types of approaches were observed beyond human samples, we applied them to environmental samples originating from seawater or sediment (Fig. 1C). In total, 32 species were found to have a mean relative abundance ≥0.5% for the sediment samples (Table 3; Table S8) and these included bacteria belonging to 23 genera. Forty-four species were identified at the same cutoff for the seawater samples (Table 3; Table S8), and these included bacteria belonging to 41 genera. A total of 30,051 and 20,519 predicted metabolites and 1,610 and 1,597 unique metabolic compounds were predicted in sediment and seawater samples, respectively, using a mean relative abundance cutoff of 0.5% (Table 3). Of note, draft metabolic models could not be built for two species in the sediment samples and a further 22 species for the seawater samples using our method due to a lack of publicly available reference genomes or representative MAGs for those species. This issue was apparent in the environmental samples as not all MAGs used for classification by MetaPhlAn4 were publicly available at the time of analysis, unlike those commonly found in human samples (19). Similar to the human samples, the number of novel predicted metabolites appeared to saturate with increasing number of input taxa (Fig. S1B).

**TABLE 3:** Microbial community metabolic modeling in environmental samples for the reference-guided and MAG-guided approaches^*a*^

Sample	Parameter	Reference-guided				MAG-guided		
		5%	2.5%	1%	0.5%	High	High and medium	Normalized
Sediment	Taxa	2 (3)	8 (9)	19 (20)	30 (32)	51	131	30
	Total predicted metabolites	2,192	8,010	20,147	30,051	46,185	120,790	27,813
	Total extracellular metabolites	102	162	193	198	200	211	193
	Unique metabolites	1,301	1,463	1,560	1,610	1,646	1,716	1,621
Seawater	Taxa	2 (3)	6 (7)	13 (23)	21 (44)	75	158	21
	Total predicted metabolites	1,835	5,930	13,391	20,519	68,130	136,462	18,331
	Total extracellular metabolites	93	167	185	195	214	216	181
	Unique metabolites	1,098	1,433	1,569	1,597	1,687	1,728	1,498

^*a*^ Summary includes the number of input bacterial species used for the metabolic predictions for each sample type (taxa), the total number of predicted metabolites (duplicates not removed), total number of metabolites classified as extracellular (duplicates not removed), and the number of non-duplicated total predicted metabolic products (unique metabolites). Numbers in brackets represent actual species detected at the relative abundance cutoff as opposed to true number of species used as input due to absence of genomic information. Normalized refers to metabolite predictions when the total number of input MAGs was matched to number of input taxa at the 0.5% cutoff.

A total of 51 high-quality MAGs and 131 high- or medium-quality MAGs and 75 high-quality MAGs and 158 high- or medium-quality MAGs were assembled from sediment and seawater samples, respectively (Tables S9 and S10). In total, the analysis included MAGs from 84 genera for the sediment samples and 86 genera for the seawater samples. In contrast to the human samples, a higher number of total and unique metabolites were observed using the MAG-guided approach compared to the reference-guided approach for the environmental samples. A total of 120,790 and 136,462 predicted metabolic compounds and 1,716 and 1,728 unique metabolic compounds were predicted in the sediment and seawater communities, respectively (Table 3). Metabolite predictions from individual taxa inputted into the models, including percentage of blocked reactions, are provided in Table S11.

To assess if this may be due to the >4- and 7-fold input in species-level taxa for the MAG-guided approach, we normalized the number of input MAGs to the number of input species in the 0.5% cutoff (sediment: 30; seawater: 21), selecting the most complete high-quality MAGs. For seawater samples, the total number (20,519 vs 18,331 metabolites) and number of unique predicted metabolites (1,597 vs 1,498 metabolites) were higher for the reference-guided approach than the MAG-guided approach (Table 3). While the total number of predicted metabolites was higher for the reference-guided approach in the sediment samples (30,051 vs 27,813 metabolites), the number of unique metabolites from the MAG-guided approach was higher (1,621 metabolites) than the reference-guided approach (1,610 metabolites) (Table 3).

**Fig 3:**
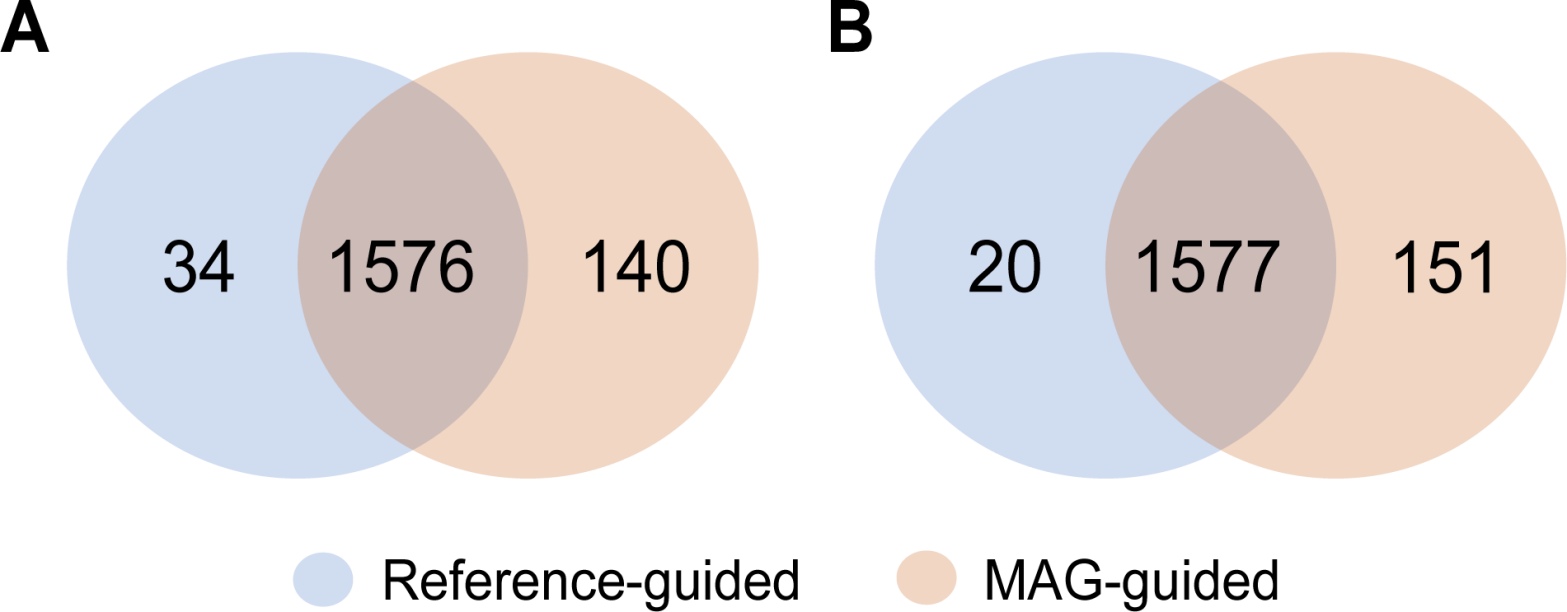
Number of unique predicted metabolic compounds across the reference-guided (0.5% cutoff) and MAG-guided (high- and medium-quality) inputs for environmental samples. (**A**) Sediment. (**B**) Seawater.

We then assessed if the approaches were complementary or additive. For the sediment samples, there was substantial overlap across the approaches (1,576 metabolites), but each approach also identified unique metabolites not detected in the other (reference-guided: 34; MAG-guided: 140 metabolites) (Fig. 3; Fig. S3). Similarly, for the seawater samples, 1,577 metabolites overlapped across the approaches, with a further 20 unique to the reference-guided approach and 151 metabolites unique to the MAG-guided approach (Fig. 3; Fig. S3).

### Effect of gapfilling on predictions of metabolic output in human and environmental samples

Next, we examined if gapfilling of the GEMs alters the conclusions from the human fecal and the environmental samples. Gapfilling increased the total number of predicted metabolites and the number of unique metabolites across both the reference-based and MAG-based approaches in both donors (Tables S4, S11 .and S12). When comparing the fold increase following gapfilling between the reference-based approach (0.5% cutoff) and the MAG-based approach (normalized), gapfilling had a similar effect on the reference-based and MAG-based approaches (donor 1: 1.05-fold vs 1.03-fold; donor 2: 1.04-fold vs 1.04-fold) and number of unique (donor 1: 1.04-fold vs 1.02-fold; donor 2: 1.02-fold vs 1.02-fold) predicted metabolites. The total number of unique predicted metabolites remained higher in the reference-based approach than the MAG-based approach in both donors (donor 1: 1,558 vs 1,536 metabolites; donor 2: 1,599 vs 1,574 metabolites; Table S12).

In the environmental samples where the reference-based approach predicted more metabolites than the MAG-based approach prior to gapfilling (when numbers of input taxa were normalized), the conclusions did not change following gapfilling, with an increase in predicted metabolites across all analyses (Table S12). Gapfilling had a similar effect (by fold-change of metabolites) on the reference-based models (0.5% cutoff) as the MAG-based models (normalized) for total number of predicted metabolites (sediment: 1.07-fold vs 1.05-fold; seawater: 1.02-fold vs 1.05-fold) and unique metabolites (sediment: 1.03-fold vs 1.03-fold; seawater: 1.02-fold vs 1.03-fold) (Table S12).

## DISCUSSION

Elucidating the metabolites produced by microbial communities is key to understanding their impact on their ecological niches and understanding their overall contribution to host metabolism and environmental processes (21–23). Although untargeted profiling of the metabolome allows for the detection of thousands of metabolites from a given sample, output is dependent on extraction protocols and the type of detection technique used, and can include metabolites not produced by the community (e.g., host). An alternative practice has been to generate draft reconstructions and model metabolic outputs by the microbial communities from metagenomic sequencing data. This is commonly performed by using MAGs binned from the data as input. However, this strategy remains computationally intensive, and it requires deep shotgun sequencing for efficient MAG binning. There has been an increase in the number of genomes for bacterial species that are available in public databases, and this provides an opportunity to leverage these genomes for construction of GEMs. Here, we systematically compared drafting GEMs using reference-guided and MAG-guided approaches for both human and environmental samples and tested our findings using untargeted metabolomics. We showed that while the reference-guided approach initially predicted less total number of metabolites than the MAG-guided approach for the human samples, it predicted more than the MAG-guided approach when the number of input taxa was normalized. The validation findings suggested that both approaches had a similar level of error. The two approaches appeared to be complementary, but despite this substantial overlap, each predicted the production of unique metabolites. Our data also suggested that biological inferences can change based on the approach used, and thus, there may be utility in integrating both approaches or validating one approach with the other. In environmental samples, similar results were observed where the MAG-guided approach predicted production of more metabolites which was due to the higher input of genome bins into this approach relative to the reference-guided approach. Despite this, the contribution of metabolic uniqueness of the input taxa cannot be discounted as an influencer of non-redundant metabolite predictions in a community GEM given the predictions of unique metabolites in sediment samples. In this sample type, the two approaches were also found to be complementary showing substantial overlap but each identified unique metabolites.

There are several strengths and limitations to the reference-guided approach that differentiate it from the MAG-guided approach. One strength includes the lack of requirement for MAG binning and refinement which is a computationally intensive process and one that remains the subject of research, specifically into accuracy of the assembled and refined genome bins (15). Through the use of genomes from pure isolates (i.e., no contamination during assembly), it can be speculated that this approach would provide more precise tracking of metabolite origin to specific taxa. The lack of need for MAGs also allows for the construction of GEMs from shallow shotgun sequencing data sets which are becoming more popular and more readily available as sequencing costs decrease. In contrast, one key limitation of the reference-guided approach was seen in the environmental samples where the reliance on reference genomes that were not readily available posed a problem. With the increase in publicly available genomes and MAGs from different species, this limitation should potentially become less of an issue; however, it is important to note that we could not obtain neither a reference genome nor a high- or medium-quality MAG for taxa that were detected at >5% relative abundance in sediment and seawater, indicating that additional manual curation of environmental MAGs is required due to known difficulties in assembly of some highly abundant organisms (24, 25). Another limitation of the reference-guided approach worth mentioning is the lack of specificity of its input to the strains within the sequenced samples, and thus, bacterial strain variations would be unaccounted for, leading to possible false predictions. However, integration of our GEM predictions with the untargeted metabolomics showed a similar percentage of validated metabolites relative to total predicted in the reference-guided approach. Lastly, when we attempted to lower the relative abundance cutoff to 0.1%, several taxa were found to be unclassified at the species level (data not shown), which would restrict their inclusion into the predictions. A key limitation of both approaches worth highlighting is the limited predictive potential due to lack of curation against experimental data; however, it is plausible to assume that curation would be more readily possible for reference genome-based draft reconstructions.

In conclusion, our work compares alternative approaches to GEM construction from metagenomes, showing utility in genomic input guided by reference-based taxonomy, which can complement current MAG-guided methods for deep shotgun metagenomics data. We demonstrate that the choice of approach alters biological inferences, emphasizing the need for caution when relying on model predictions. Notably, this reference-guided approach could also be applied to shallow sequencing data where MAGs cannot be generated.

## MATERIALS AND METHODS

### Human stool samples and generation of MAGs

The collection of stool samples from healthy individuals was part of a study on fecal microbiota transplantation in the treatment of ulcerative colitis (20), of which the healthy individuals were donors of the study (donors 1 and 2).

A total of 30 fecal samples were collected from two healthy individuals over a period of 44 and 70 weeks (Fig. 1B). DNA was extracted from the lyophilized material using the QIAamp PowerFecal DNA Kit (Qiagen, Chadstone, Victoria, Australia) and sequenced on the Illumina NovaSeq 6000 (S4 2 × 150 bp) using the Illumina DNA prep kits (Illumina, Melbourne, Victoria, Australia) as previously reported (26). Raw sequencing reads belonging to the same sample were concatenated into a single set of forward and reverse fastq files and were then quality trimmed using the Read_qc module to trim adaptors and remove human contamination. Reads were assembled using MetaBAT v.2 (27), MaxBin v.2 (28) and CONCOCT within MetaWRAP v.1.3.2 with default parameters. MAGs were generated with default parameters and subsequently refined using MetaWRAP v.1.3.2 (29). The completeness and contamination of MAGs were determined with CheckM (30). MAGs were defined as “high-quality” when they were >90% complete with less than 5% contamination, or “medium-quality” when they had a completeness of ≥50% and less than 10% contamination (31). MAGs that were “high-quality” or “medium-quality” were then dereplicated at 99% average nucleotide identity using dRep v.2.3.2 to remove duplicate MAGs (32). Taxonomy was assigned to each MAG based on the Genome Taxonomy Database (GTDB) r207 (33) with GTDB-Tk v.1.5.1 (34).

### Environmental samples and generation of MAGs

Sediment (*n* = 10) and seawater (*n* = 10) samples were taken from coastal locations in Sydney and Perth, Australia (Fig. 1C). DNA was extracted from seawater and sediment samples as per standard operating procedures (https://github.com/AusMicrobiome/scientific_manual) and sequenced on a NovaSeq 6000 sequencer (2 × 150 bp run) using the Illumina DNA prep kit at the Ramaciotti Centre for Genomics (UNSW Sydney, Australia). Raw sequencing reads belonging to the same sample were concatenated into a single set of forward and reverse fastq files. Reads were then quality trimmed with Trimmomatic v.0.38 (35) with parameters specified as “HEADCROP:10 SLIDINGWINDOW:4:30.” Seawater samples were assembled with metaSPAdes v.3.15.0 (36) with k-mer options 21,41,61,81,101,121,127, while sediment samples were assembled using MEGAHIT v.1.2.2b (37) with k-mer options specified as –k-min 21 –k-max 141 –k-step 20. Scaffolds smaller than 2,000 bp and 2,500 bp were removed for sediment and seawater samples, respectively. The coverage of the remaining scaffolds was determined by mapping of the quality-filtered reads using bowtie v.2.3.5.1 (38). After converting and sorting the mapping format using samtools (39), the coverage was determined with the jgi_summarize_bam_contig_depths script (27). MAGs were generated using MetaBAT v.2.12.1 (27) and MaxBin v.2.2.3 (28) with default parameters, and subsequently refined using MetaWRAP v.1.3 (29). The completeness and contamination of MAGs were determined with CheckM, and MAGs were defined as “high-quality” or “medium-quality” as above. MAGs that were “high-quality” or “medium-quality” were then dereplicated and taxonomically assigned as above.

### Generation of species-level count tables for reference-guided approach

Quality-filtered reads from above were analyzed using MetaPhlAn4 to profile the composition of the microbial communities and generate tables with relative abundances of microbial species (40). The mean relative abundances of the microbial species within each sample group (i.e., donor 1, donor 2, seawater, and sediment) were calculated. Microbial species were grouped according to the cutoffs of ≥5%, ≥2.5%, ≥1%, and ≥0.5% mean relative abundance for this approach.

### Construction of the genome-scale metabolic models

Genomes/MAGs were annotated in KBase with default parameters using the Annotate Genome/Assembly with RASTtk v.1.073 App prior to building the draft metabolic models for each organism. This included a similarity e-value cutoff of 1e−06. GEMs were built using MS2–Build Prokaryotic Metabolic Models with OMEGGA App implemented in KBase based on the ModelSEED Pipeline for individual genomes or MAGs. Models for genomes and MAGs classified as *Synechococcus* could not be built with the MS2–Build Prokaryotic Metabolic Models with OMEGGA App and therefore were built using the Build Metabolic Model App (41, 42). Based on this method, biomass components of species are included within reactions if their genome contains the appropriate subsystems and annotations (42). All reactions associated with enzymes encoded in the annotated genome are included in the models, with spontaneous reactions also added (42). Draft models from individual organisms were then merged into a joint model of a community of multiple organisms using the app “Merge two or more metabolic models into a compartmentalized community model.” In this joint multi-species model, compounds and reactions in each species are placed in uniquely labeled compartments, with compounds transported out of any member placed into a shared extracellular environment that can be accessed by any member possessing a transport reaction that can import an available extracellular compound (41, 42).

For the MAG-guided models, high-quality MAGs and high- and medium-quality MAGs were used to construct the GEM. For the reference-guided models, the relative abundance of bacteria was calculated, and input taxa were selected based on different cutoffs (i.e., ≥5%, ≥2.5%, ≥1%, and ≥0.5%). Genomic information from the selected bacteria were then employed to construct the GEMs. Representative genomes for the reference-guided approach were downloaded from the KBase public database and, if not present there, were downloaded directly from NCBI. A total of 15, 27, 24, and 20 taxa had only one genome available for donor 1, donor 2, sediment, and seawater samples, respectively. Where more genomes were available, the genome with the highest number of features was chosen. This strategy was implemented to maximize metabolic output from the selected reference genomes. However, comparison of predictions from genomes with the highest number of features with those with the lowest number of features in the same bacterial species showed that this increased the total number of predictions in 15 of the 19 species in human samples where multiple genomes were available (Table S13). In contrast, four outputs decreased (Table S13). For environmental samples, this strategy resulted in increased, similar, or decreased predictions for three, one, and three species where multiple genomes were available (Table S13). In certain cases where a public genome was not available (some taxa classified by MetaPhlAn4 to species-level genome bins), the corresponding MAGs present within the data were uploaded and merged into the community model. Not all species that were selected for the reference-guided approach in the environmental samples could be included as neither reference genomes nor MAGs were publicly available, and this was attributed to difficulties in assembly of highly abundant organisms.

To report the outputs of the model, values for “total predicted metabolites,” “total extracellular metabolites,” and “unique metabolites” were provided. Total predicted metabolites refer to all metabolites present in a system (all compartments, duplicates not removed). Total extracellular metabolites refer to metabolites present in a system classified as extracellular (duplicates not removed). Unique metabolites refer to the total predicted metabolites (all compartments) filtered for redundancy, in an effort to reflect metabolic diversity.

To assess its effect on metabolite predictions, all individual models for the reference-guided and MAG-guided approaches were gapfilled using the app MS2–“Improved Gapfill Metabolic Models with OMEGGA” and then merged into a compartmentalized community model. Flux balance analysis (FBA) was applied on gapfilled metabolic models for both the reference-guided and MAG-guided approaches using the app Run Flux Balance Analysis. The Run FBA method uses flux variability analysis (43) to classify if the reactions in the models are unable to carry flux (i.e., blocked).

### Untargeted metabolomics from human stool samples

Untargeted metabolomics analysis on stool samples from the healthy individuals was performed using Metabolon’s Precision Metabolomics liquid chromatography-mass spectrometry global metabolomics platform and was previously reported (26). These were included to validate the output inferences from the GEMs. The samples analyzed were collected from the individuals on weeks 24, 26, 34, and 40 for donor 1 and weeks 20, 22, 31, 33, 41, 42, and 70 for donor 2 (Fig. 1B), and hence, the samples overlap longitudinally and extensively with the metagenomics data, overcoming biological variability associated with cross-sectional sampling. Raw metabolite abundance data prior to imputation was converted to presence/absence of metabolite per donor. Experimentally validated metabolites were reported as “confirmed unique metabolites.”

For donor 1, a total of 1,348 metabolites were identified by untargeted metabolomics, of which 489 had KEGG IDs. An additional 34 KEGG IDs corresponded to the same metabolites (replicate IDs) and were included in the searches, making 523 the total number of KEGG IDs for donor 1. For donor 2, a total of 1,190 metabolites were identified by untargeted metabolomics, of which 453 had KEGG IDs. An additional 35 KEGG IDs corresponded to the same metabolites (replicate IDs) and were included in the searches, making 488 the total number of KEGG IDs for donor 1. It is important to note that fecal metabolomics is not restricted to bacterial metabolites and will include human metabolites and those from other members of the microbiota.

### Pathway enrichment analysis of predicted metabolites

Following GEM construction, metabolite lists were imported into MBROLE 2.0 to perform pathway enrichment analyses (44) using pathways from the Small Molecule Pathway Database (human), KEGG, and UniPathway. MBROLE annotates the metabolites in the test and background lists with their respective pathways within the selected databases, then performs an over-representation analysis using cumulative hypergeometric distribution, after which the *P*-values are corrected for false discovery rate using the Benjamini and Hochberg method (45).

## Data Availability

The human raw metagenomics reads are available from the European Nucleotide Archive (ENA) under the accession PRJEB50699. The environmental raw metagenomic reads are available through the BioPlatforms Australia data portal (https://data.bioplatforms.com/). Table S14 includes the list of individual accession links. The KBase narratives can be accessed through the html links found in Table S15.

## SUPPLEMENTAL MATERIAL

Supplemental Figures msystems.00746-24-s0001.docx Figures S1 to S3.

Supplemental Tables msystems.00746-24-s0002.xlsx Tables S1 to S15.

ASM does not own the copyrights to Supplemental Material that may be linked to, or accessed through, an article. The authors have granted ASM a non-exclusive, world-wide license to publish the Supplemental Material files. Please contact the corresponding author directly for reuse.
