# Supplementary material for: Refining microbial community metabolic models derived from metagenomics using reference-based taxonomic profiling
Source: mSystems. 2024 Aug 13;9(9):e00746-24. doi: 10.1128/msystems.00746-24 (PMC11406951; doi:10.1128/msystems.00746-24)

**Supplementary Figure 1**

**Number of unique predicted metabolites using the reference-guided approach across all relative abundance cut-offs. A:** Human fecal samples. **B:** Environmental samples.

**
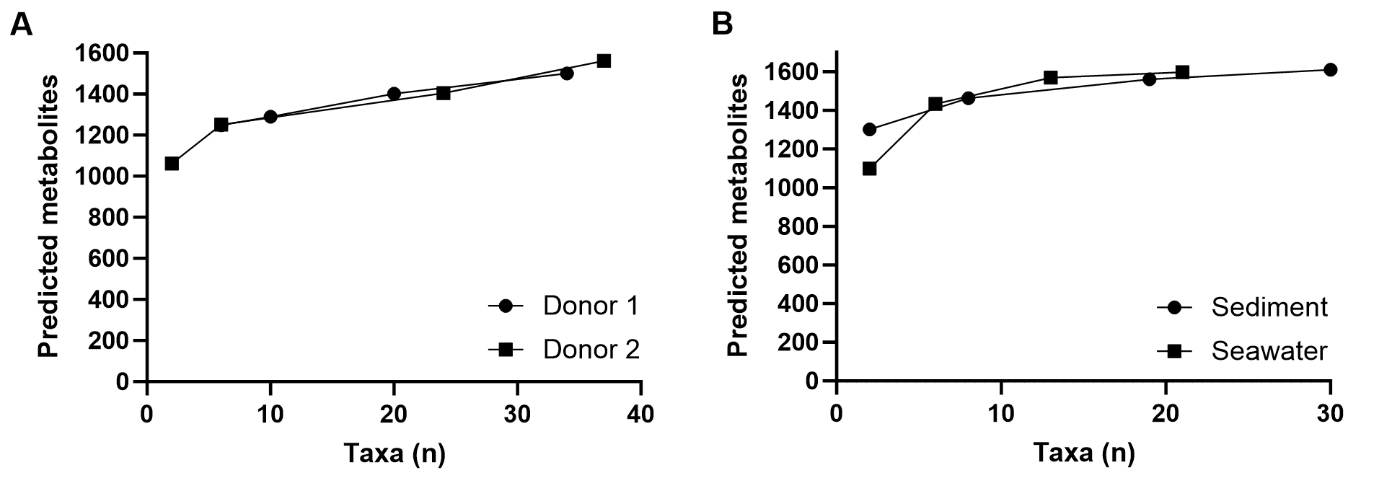
**

**Supplementary Figure 2**

**Venn diagram showing the number of unique predicted metabolic compounds across all reference-guided and MAG-guided inputs for human faecal samples. A:** Donor 1. **B:** Donor 2.


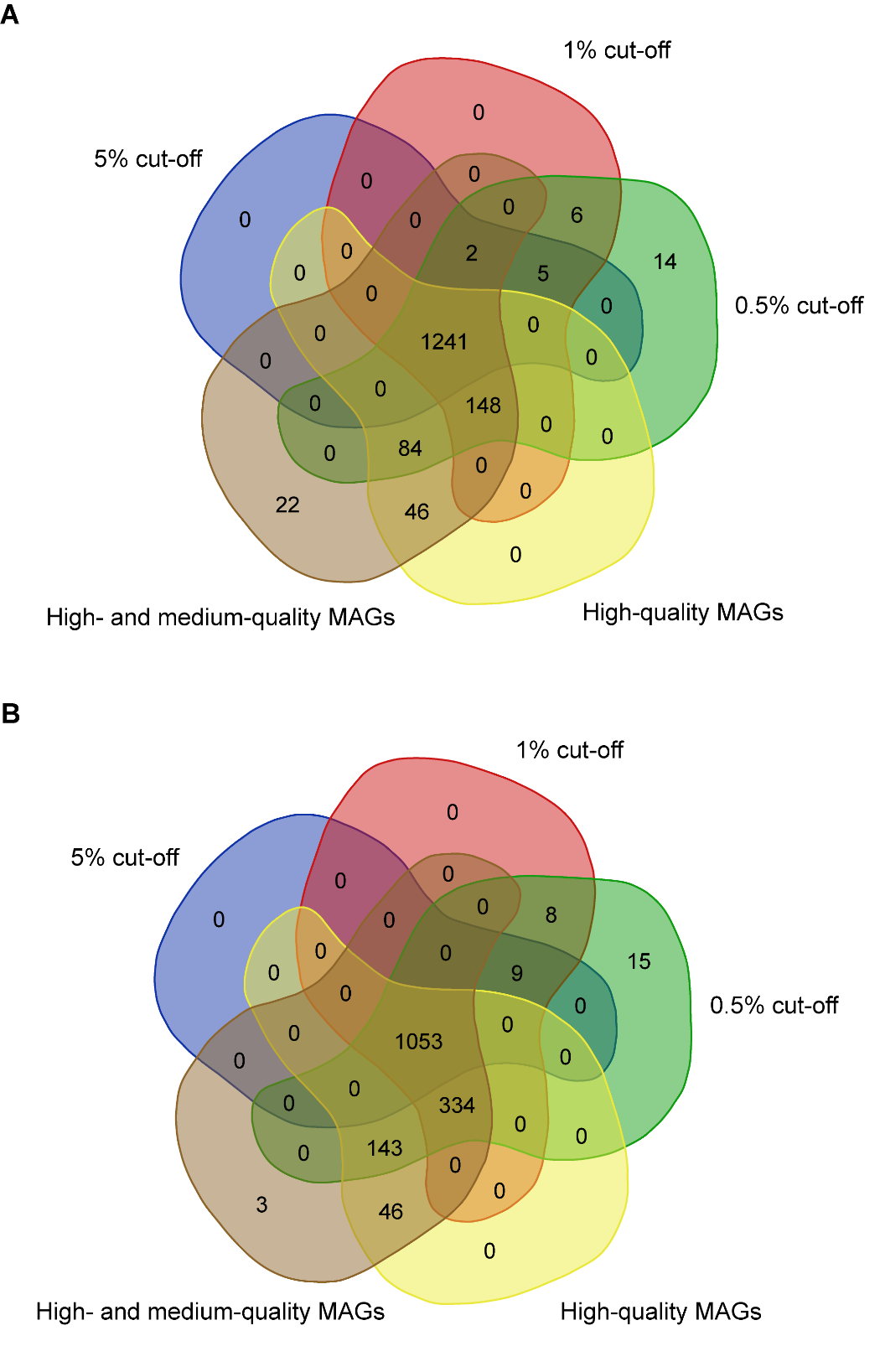


**Supplementary Figure 3**

**Venn diagram showing the number of unique predicted metabolic compounds across all reference-guided and MAG-guided inputs for environmental samples.** **A:** Sediment. **B:** Seawater.


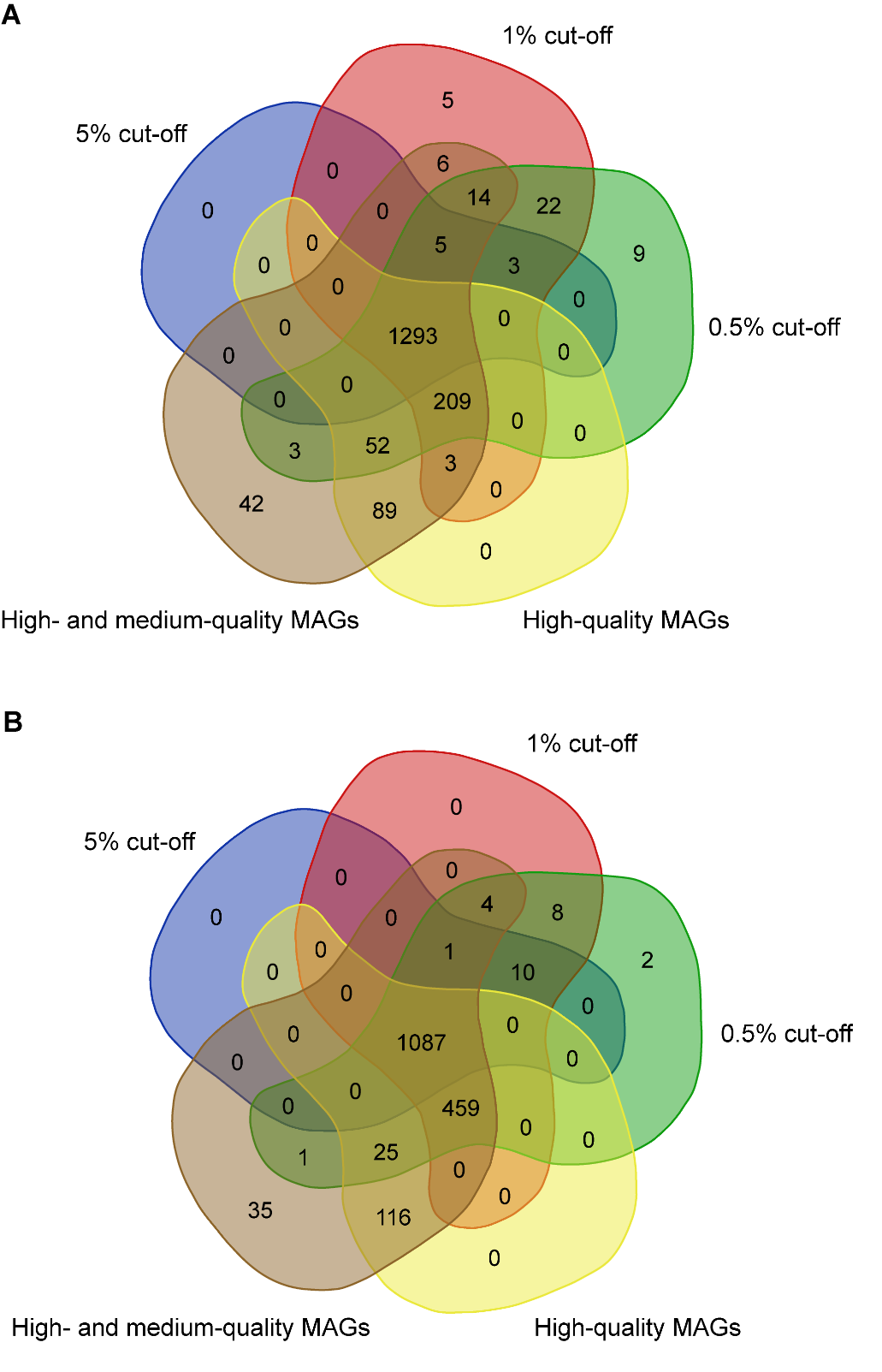

Supplement: Supplemental Figures — Figures S1 to S3. [file msystems.00746-24-s0001.docx]
